# Tenascin-C induces migration and invasion through JNK/c-Jun signalling in pancreatic cancer

**DOI:** 10.18632/oncotarget.20160

**Published:** 2017-08-10

**Authors:** Jun Cai, Shaoxia Du, Hui Wang, Beibei Xin, Juan Wang, Wenyuan Shen, Wei Wei, Zhongkui Guo, Xiaohong Shen

**Affiliations:** ^1^ School of Medicine, Nankai University, Tianjin 300071, China; ^2^ Tianjin Medical University Cancer Institute and Hospital, National Clinical Research Center for Cancer, Tianjin 300060, China

**Keywords:** TNC, JNK/c-Jun, EMT, pancreatic cancer

## Abstract

Tenascin-C (TNC), a large extracellular matrix glycoprotein, has been reported to be associated with metastasis and poor prognosis in pancreatic cancer. However, the effects and mechanisms of TNC in pancreatic cancer metastasis largely remain unclear. We performed Transwell assays to investigate the effects of TNC on Capan-2, AsPC-1 and PANC-1 cells. In addition, western blot and RT-qPCR assays were used to examine potential TNC metastasis-associated targets, such as JNK/c-Jun, Paxillin/FAK, E-cadherin, N-cadherin, Vimentin, and MMP9/2. Lastly, we utilized a variety of methods, such as immunofluorescence, gelatin zymography and immunoprecipitation, to determine the molecular mechanisms of TNC in pancreatic cancer cell motility. The present study showed that TNC induced migration and invasion in pancreatic cancer cells and regulated a number of metastasis-associated proteins, including the EMT markers, MMP9 and Paxillin. Moreover, our data showed that TNC induced pancreatic cancer cells to generate an EMT phenotype and acquire motility potential through the activation of JNK/c-Jun signalling. In addition, TNC increased the DNA binding activity of c-Jun to the *MMP9* promoter, an action likely resulting in increased MMP9 expression and activity. TNC/JNK also markedly induced the phosphorylation of Paxillin on serine 178, which is critical for the association between FAK and Paxillin and promoted the formation of focal adhesions. TNC/JNK initiates cell migration and invasion of pancreatic cancer cells through the promotion of EMT, the transactivation of MMP9 and the phosphorylation of Paxillin on serine 178. TNC may be a potential therapeutic target for treating pancreatic cancer metastasis.

## INTRODUCTION

Pancreatic ductal adenocarcinoma (PDAC) is a gastrointestinal malignancy with an extremely poor prognosis and a 5-year survival rate of less than 8% [1]. The mechanisms of the aggressive growth and metastasis are not yet thoroughly understood. A defining feature of PDAC is its dense tumour-associated stroma. Previous studies have shown that various extracellular matrix (ECM) proteins function in cancer progression and prognosis [2, 3]. However, the role of ECM proteins involved in PDAC metastatic processes still needs intensive study.

TNC is a large glycoprotein located in the extracellular matrix. It is expressed during organogenesis and regulates the interactions between the parenchyma and mesenchyme in physiological or pathological conditions accompanying cell proliferation and migration and epithelial-mesenchymal transition (EMT) [4-6]. TNC is rarely expressed in normal adult pancreatic tissue. However, it was found to be highly expressed in pancreatic cancer, and expression of TNC is correlated with cancer metastasis and the progression from low-grade precursor lesions to PDAC [7, 8]. In pancreatic cancer, both pancreatic stellate cells (PSCs) and pancreatic tumour cells can synthesize TNC and then efficiently secrete it into the stroma [7]. Thus, TNC might play a role in tumour cell progression and metastasis in pancreatic cancer, and the underlying mechanisms of such an effect needed to be verified.

Multistep processes are discernible in the biological cascade of cancer cell metastasis, such as alteration of cell biological properties and loss of cellular adhesion or increased motility and invasiveness. In addition, many molecules involved in these processes induce cancer metastasis. The c-Jun N-terminal kinase (JNK) belongs to the mitogen-activated protein kinase (MAPK) family, which is activated in response to various extracellular stimuli, such as epidermal growth factor (EGF), tumour necrosis factor (TNF), transforming growth factor β (TGF-β) and TNC [9, 10]. Activation of JNK has been reported to promote development in various cancers, including pancreatic cancer [11, 12]. JNK plays a vital role in differentiation, apoptosis and cell migration, and its oncogenic functions are mostly based on its ability to phosphorylate c-Jun and to activate transcriptional factor Activator Protein-1 (AP-1). Matrix metalloproteinases (MMPs), such as MMP1, MMP9 and MMP13, have an AP-1 consensus sequence. These degradative enzymes regulate tumour progression by enhancing tumour-induced angiogenesis and destroying local tissue architecture and basement membranes to allow tumour invasion and metastasis. Co-expression of TNC and MMP9 or MMP2 was associated with a poorer prognosis and was found to be an independent predictor of survival for pancreatic cancer patients [13, 14]. Therefore, TNC/MMPs might regulate cancer cell invasion. In addition, the EMT programme induces changes in the properties of epithelial cells and causes them to lose their polarity and gain the ability to migrate and invade during developmental morphogenesis. Recent studies have shown that EMT is involved in the invasion and metastasis of many types of carcinomas, including PDAC [15]. TNC induced EMT-like changes, such as loss of intercellular adhesion and enhanced migration in breast cancer cells [16]. However, whether TNC induces EMT in pancreatic cancer is still unclear. Moreover, JNK can modulate focal adhesion formation by regulating the FAK/Paxillin association^S178^ [17]. Therefore, we speculated that JNK signalling might play a key role in TNC-mediated motility in PDAC cells.

In this study, we present evidence that TNC activates JNK/c-Jun signalling, resulting in induction of EMT and transcriptional activation of MMP9 to enhance the metastatic properties of tumour cells. TNC can activate JNK to promote the association of Paxillin with FAK, which facilitates the motility and adhesion of pancreatic cancer cells. TNC modulates the metastatic process in the form of a biological cascade effect.

## RESULTS

### TNC promotes migration and invasion abilities of pancreatic cancer cells

**Figure 1:**
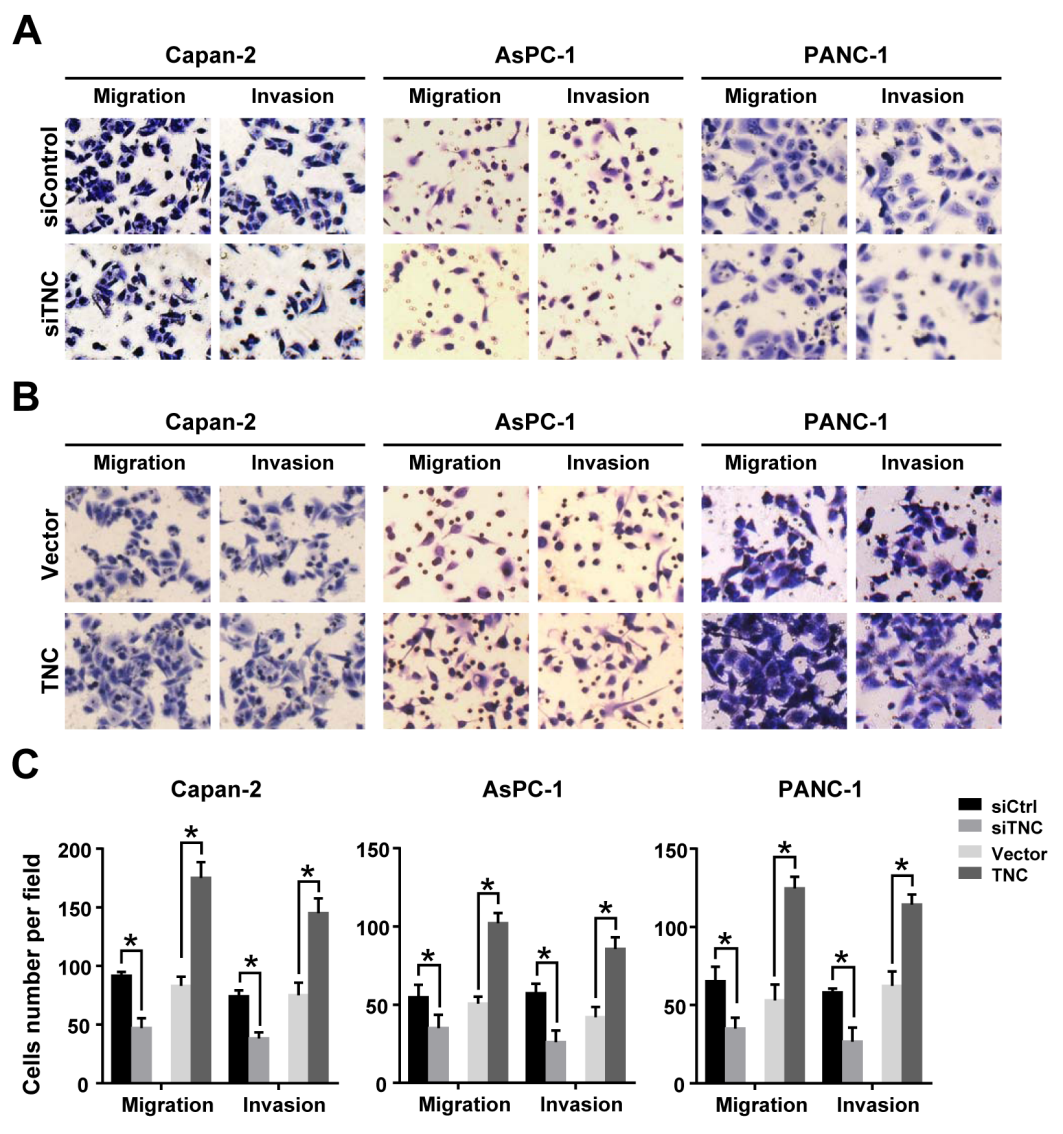
TNC promotes pancreatic cancer cell migration and invasion **(A)** Inhibition of TNC expression decreased tumour cell migration and invasion in Capan-2, AsPC-1 and PANC-1 cells. **(B)** Upregulation of TNC enhanced tumour cell migration and invasion in Capan-2, AsPC-1 and PANC-1 cells. **(C)** The number of migrated and invasive cells in pancreatic cancer cell lines transfected with siTNC or TNC plasmid. Data represent the mean ± SD. (n = 3, *P < 0.05).

TNC overexpression has repeatedly been observed in various types of tumours, especially in the front of invasive tumours. Esposito et al. reported that TNC could be synthesized by the cancer cells and then efficiently secreted in the stroma, but the protein level of TNC can’t be detected in pancreatic cell lines [7]. So we tested the mRNA and protein levels for confirming the interference/overexpression efficiency of TNC after the pancreatic cancer cells transfected with siTNC or TNC expression plasmid. (Supplementary Figure 1). To investigate whether TNC regulates migration and invasion in pancreatic cancer cells, we first knocked down the expression of TNC in Capan-2, AsPC-1 and PANC-1 cells using siRNA targeting human TNC mRNA. Then, we performed Transwell assays, and the results showed that TNC depletion markedly reduces the migration and invasion abilities of all three pancreatic cancer cell lines (Figure 1A). In addition, the migration and invasion abilities were significantly increased in TNC-overexpressing cells compared with control cells (Figure 1B). The data suggested that TNC contributes to the aggressive potential of pancreatic cancer cells.

### TNC modulates the expression of EMT markers and MMP9

**Figure 2:**
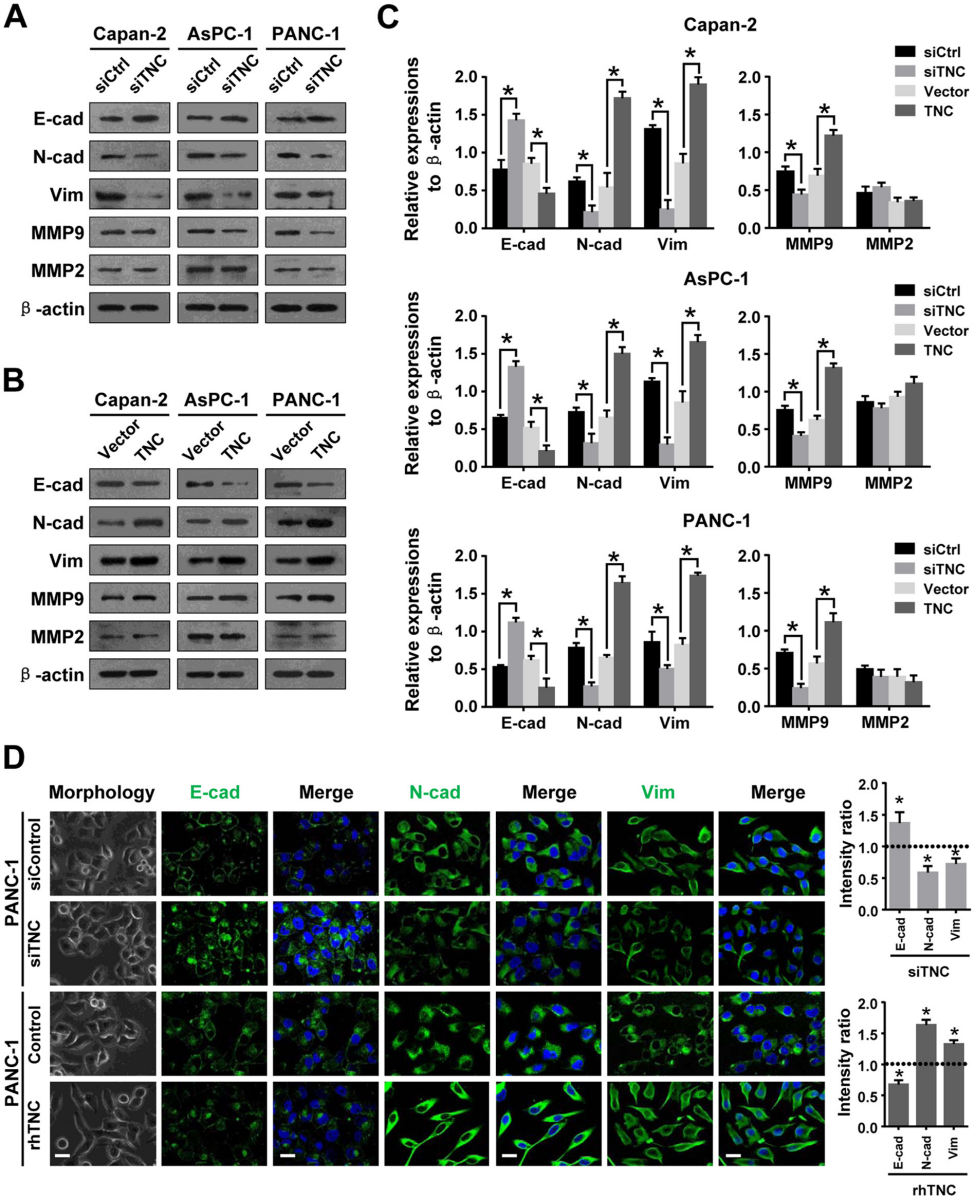
TNC regulates tumour metastasis-related genes in pancreatic cancer cells **(A)** Western blot analysis was performed to test the expression of epithelial (E-cadherin) and mesenchymal markers (N-cadherin, Vimentin) and MMPs (MMP9 and MMP2) in the Capan-2, AsPC-1 and PANC-1 cells transfected with siTNC or siControl. **(B)** The protein levels of EMT markers and MMPs were detected by western blot in Capan-2, AsPC-1 and PANC-1 cells after transfection with TNC plasmid or control vector. **(C)** The protein levels of E-cadherin, N-cadherin, Vimentin, MMP9 and MMP2 were normalized to β-actin in Capan-2, AsPC-1 and PANC-1 cells after the indicated treatment. **(D)** Cell morphology and confocal microscopy images of PANC-1 cells transfected with siTNC or coaded with rhTNC, as well as their controls. The green signal represents staining corresponding to E-cadherin, N-cadherin or Vimentin, and the blue signal represents the nuclear DNA staining by DAPI. Scale bar, 20 μm. The fluorescent intensity was determined and standardised to the cellular background. The levels of E-cad, N-cad and Vim were calculated as a ratio with compared to the controls. Data represent the mean ± SD.

To determine the mechanism underlying the promotion of migration and invasion induced by TNC, we detected the expression of tumour metastasis-related genes by western blot and immunofluorescence analysis. As shown in Figure 2A, TNC-ablation induced a more cobble stone-like shape typical of epithelial cells, which manifested an increased cell-to-cell adhesion. Consistent with the phenotypic change associated with TNC-depletion, an increased expression of the epithelial marker E-cadherin (*CDH1*), concomitant with a downregulation of the mesenchymal markers N-cadherin (*CDH2*) and Vimentin (*VIM*), (Figure 2A) was observed. These changes in EMT phenotype were also verified by an immunofluorescence assay (Figure 2D). However, ectopic expression of TNC in pancreatic cancer cells resulted in downregulation of E-cadherin and upregulation of N-cadherin and Vimentin, as evidenced by western blot analysis (Figure 2B) and immunofluorescence assay (Figure 2D). During the culture of these cells, we noticed that PANC-1-rhTNC cells presented a more spindle-like cell shape and a scattered distribution (Figure 2D), indicating that these cells may be undergoing EMT. These findings suggested that TNC can induce EMT and may be associated with an aggressive phenotype in pancreatic cancer cells.

It has been reported that coexpression of MMP9 and TNC is significantly associated with pancreatic cancer metastasis [13]. Several studies have also indicated a role for TNC in regulating the expression of MMPs [10, 18]. Therefore, we next investigated a possible mechanism by which TNC regulates MMP9 expression. The results showed that downregulation of TNC decreased MMP9 expression, whereas upregulation of TNC increased MMP9 expression in pancreatic cancer cells. We speculated that TNC might regulate MMP9 expression and thus be involved in metastatic processes in pancreatic cancer, but we found that the expression of TNC did not affect MMP2 expression in these cells.

### TNC affects cell migration and invasion through activation of JNK signalling

**Figure 3:**
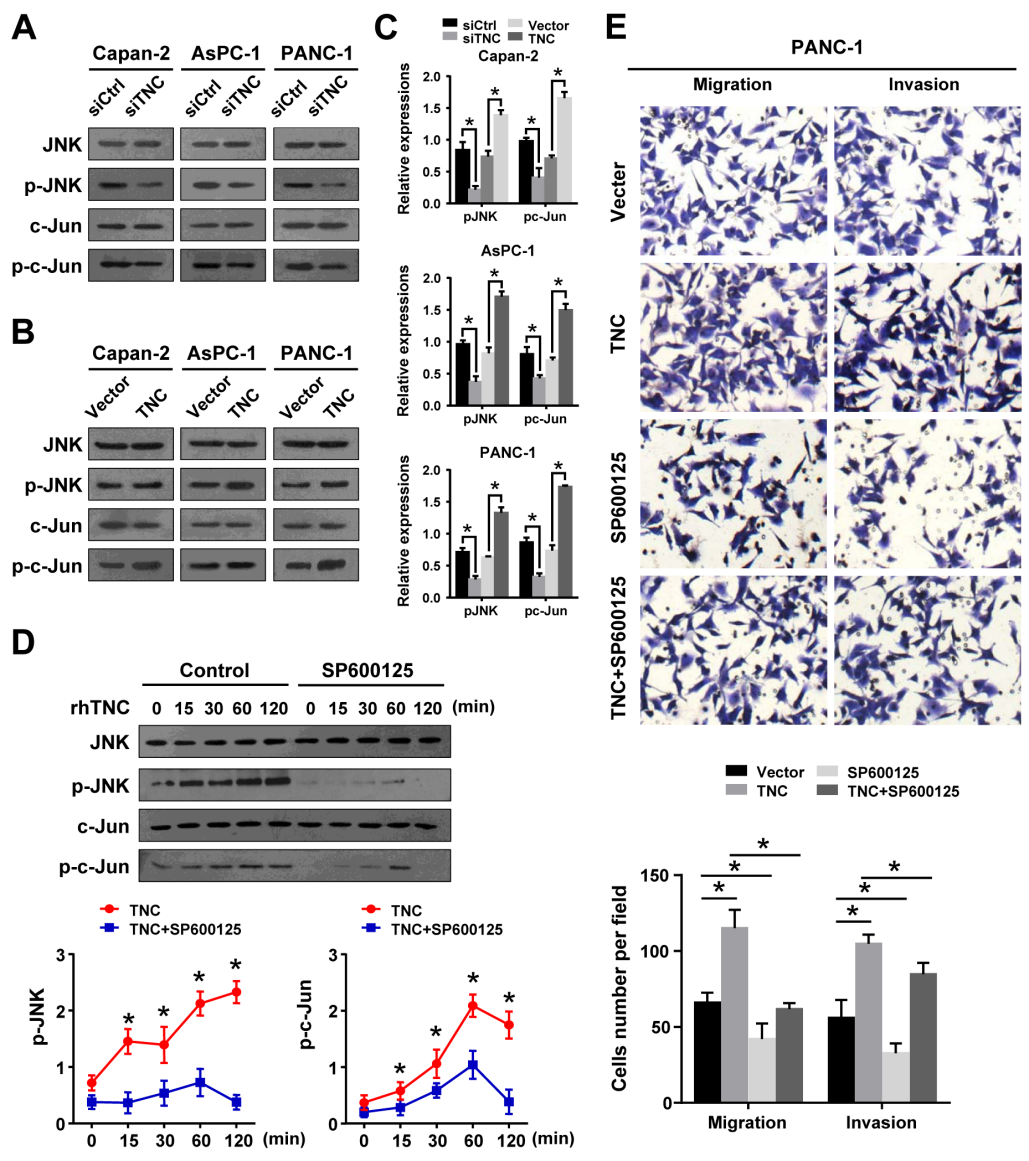
JNK signalling mediates TNC regulation of cell migration and invasion **(A)** A western blot analysis was performed to test the phosphorylated and total levels of JNK and c-Jun in the Capan-2, AsPC-1 and PANC-1 cells transfected with siTNC or siControl. **(B)** The total and phosphorylated protein levels of JNK and c-Jun were analysed by western blot in Capan-2, AsPC-1 and PANC-1 cells after treatment with TNC plasmid or control vector. **(C)** The expression levels of p-JNK and p-c-Jun were normalized to the total JNK and c-Jun levels. **(D)** PANC-1 cells were preincubated with SP600125 or DMSO for 1 h before exogenous TNC stimulation. Cells were harvested at the indicated time, and the levels of total and phosphorylated JNK and c-Jun were analysed by western blot. **(E)** PANC-1 cells were preincubated with SP600125 for 1 h before transfection with TNC plasmid or control vector. Then, the cell migration and invasion capacities were assessed using Transwell assays. Data represent the mean ± SD. (n = 3, *P < 0.05).

Several studies have reported that the JNK signalling pathway is associated with the expression of EMT markers and MMPs [19, 20]. To test the underlying mechanisms by which TNC affects PDAC metastasis, we knocked down or overexpressed TNC in Capan-2, AsPC-1 and PANC-1 cells and analysed the protein expression of total and phosphorylated JNK and c-Jun by western blot. After knockdown of TNC in tumour cells, the expression of phosphorylated JNK and c-Jun were significantly decreased compared to siControl cells (Figure 3A). However, overexpression of TNC in these cells increased the phosphorylation of JNK and c-Jun (Figure 3B). To further evaluate whether exogenous TNC can active the JNK/c-Jun signalling pathway, PANC-1 cells were exposed to exogenous TNC for various periods of time, and the subsequent phosphorylation of JNK and c-Jun were analysed by western blot. As shown in Figure 3D, stimulation of PANC-1 cells with exogenous TNC markedly increased the phosphorylation of JNK and c-Jun, and those effects were shown to be time dependent. Notably, the phosphorylation of JNK and c-Jun reached a peak (approximately six-fold) 1 h after TNC treatment, but this effect was significantly suppressed by the JNK inhibitor SP600125. The results suggested that TNC activates JNK signalling in pancreatic cancer cells. Furthermore, to explore the role of JNK activation in TNC-induced migration and invasion, we examined the effect of TNC on the aggressive potential of PANC-1 cells in the presence or absence of SP600125. The Transwell assay showed that the enhancement of the aggressive potential induced by TNC was significantly suppressed by SP600125 (Figure 3E). The data indicated that the JNK signalling pathway mediated TNC-regulated aggressive behaviour in pancreatic cancer cells.

### TNC induced EMT by regulating JNK signalling

**Figure 4:**
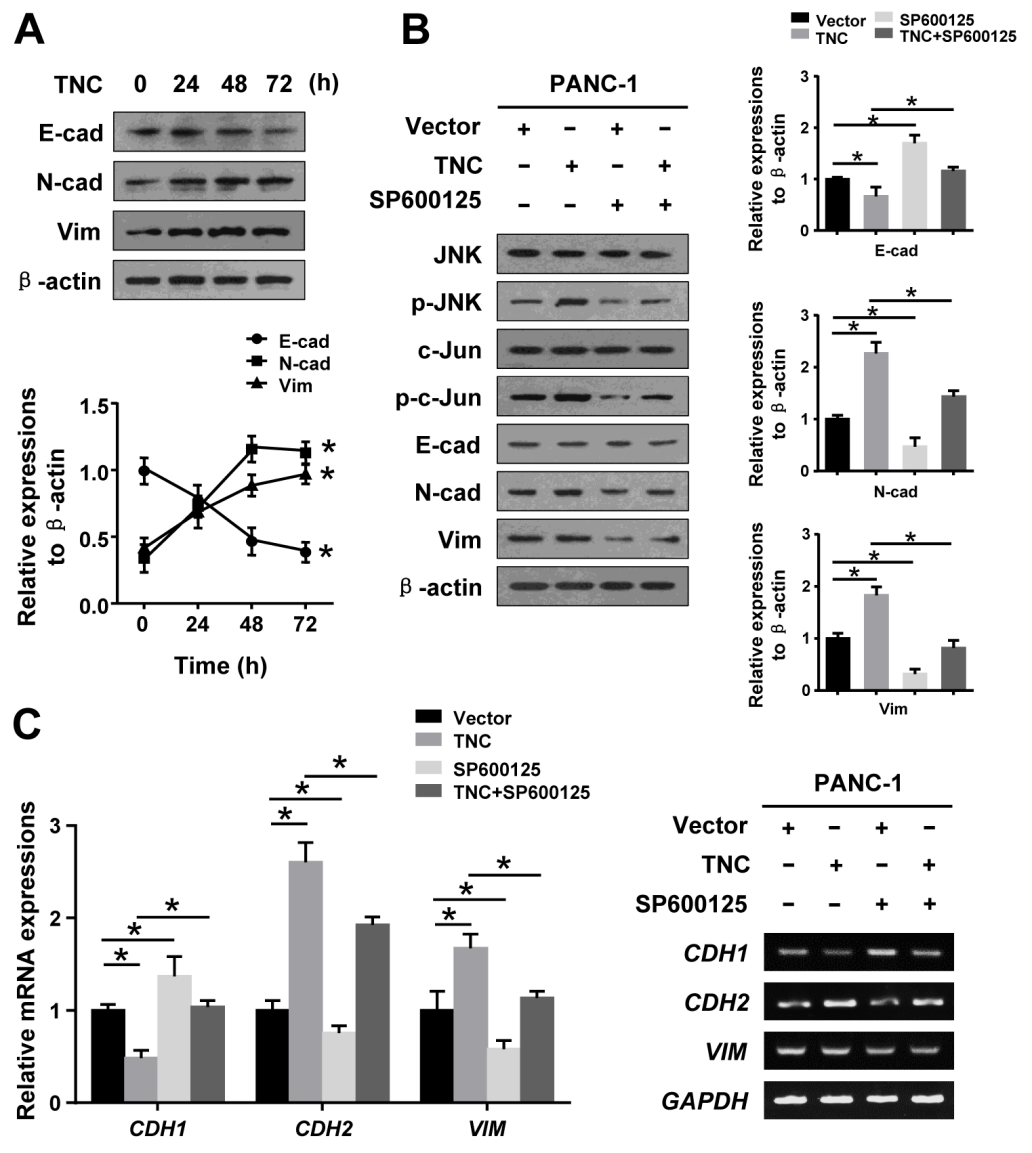
TNC regulated EMT by activating JNK signalling **(A)** PANC-1 cells were transfected with TNC plasmid for 24 h, 48 h and 72 h, and then, the protein expression levels of EMT markers were assayed by western blot. **(B)** PANC-1 cells were incubated for 1 h with SP600125 prior to being transfected with TNC plasmid or control vector, and the lysates were harvested after 48 h. The protein levels of total and phosphorylated JNK signalling proteins and EMT markers were assayed by western blot. **(C)** The mRNA levels of EMT markers in the indicated cells were measured by RT-qPCR. Data represent the mean ± SD. (n = 3, *P < 0.05).

In the present study, we have shown that TNC induces EMT alterations in Canpan-2, AsPC-1 and PANC-1 cells. We further tested the regulation effect of TNC on EMT initiation and progression in tumour cells. We found that N-cadherin and Vimentin were upregulated, and the expression peaked at 48 h and was maintained for 72 h. However, E-cadherin expression was decreased after TNC treatment in PANC-1 cells (Figure 4A). Additionally, based on the above results, we propose that TNC is an important modulator for inducing phosphorylation of JNK to initiate EMT in pancreatic cancer cells. Therefore, we examined alterations in the expression of EMT markers in PANC-1 cells after treatment with TNC and/or SP600125 and the corresponding control conditions. Western blot and RT-qPCR results show that TNC upregulated p-JNK, p-c-Jun, N-cadherin and Vimentin and decreased E-cadherin expression in PANC-1 cells, whereas SP600125 significantly reversed the activity of TNC, which suggested that TNC mediates EMT in PANC-1 cells by activating the JNK/c-Jun signalling pathway (Figure 4B and 4C).

### TNC transactivates MMP9 expression via activation of c-Jun

**Figure 5:**
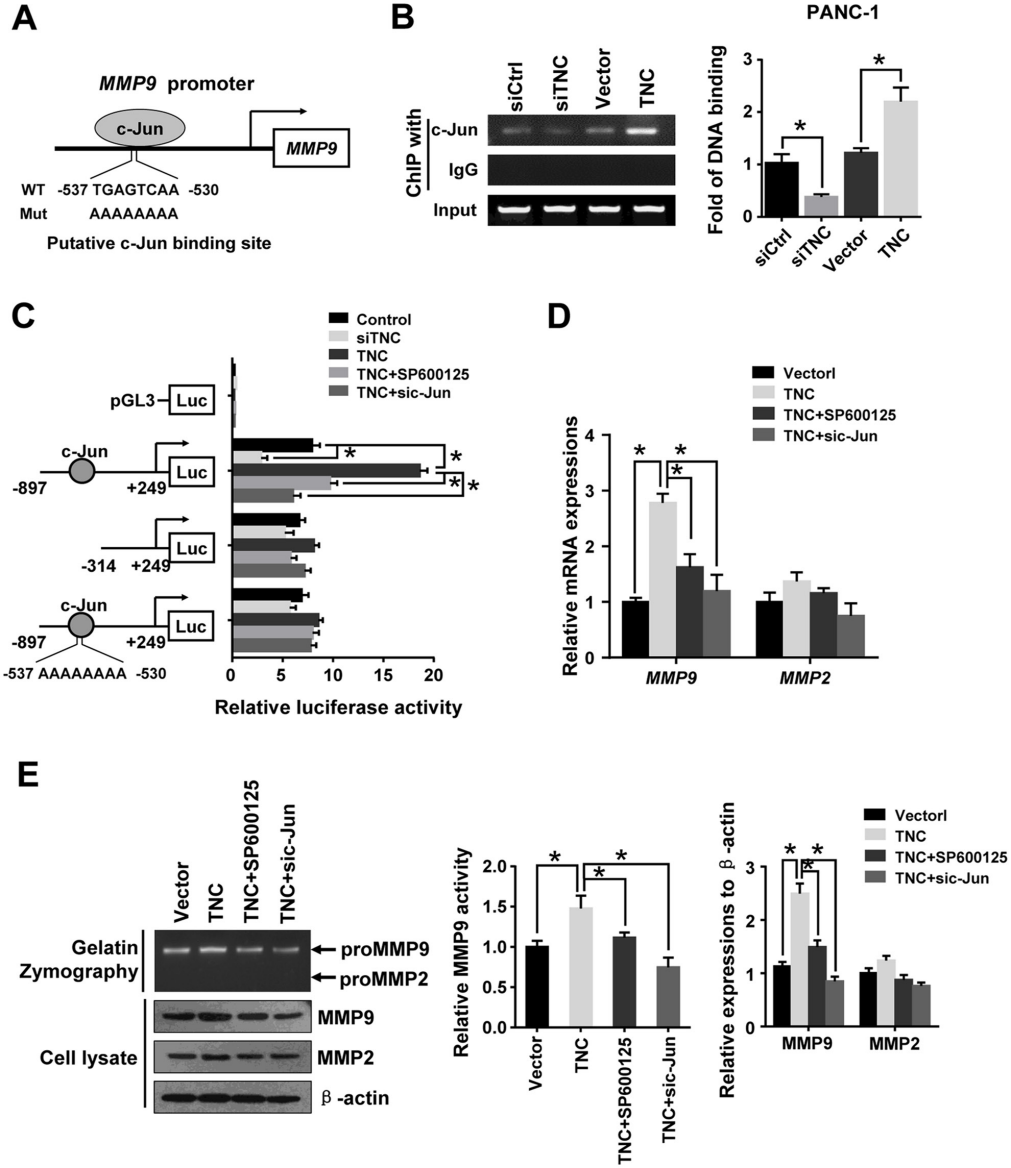
C-Jun was required for TNC-induced MMP9 expression **(A)** The sequence and position of the c-Jun binding site in the *MMP9* promoter are shown. **(B)** ChIP of c-Jun on the promoter of *MMP9*. PANC-1 cells were transfected with empty siCtrl, siTNC, vector or TNC plasmid. PCR amplification from the total chromatin (bottom) was used as a positive control, anti-IgG (middle) served as a negative control, and anti-c-Jun (top) showed the interaction between the c-Jun and *MMP9* promoter after the indicated treatment. **(C)** TNC transactivates the *MMP9* promoter via c-Jun. The luciferase activity of the reporters in the indicated cells was assessed by a dual-luciferase reporter assay. The relative luciferase activity is the ratio of the luciferase activity in each of the tested cells to that in the control cells. Data represent the mean ± SD. (n = 3, *P < 0.05). **(D, E)** PANC-1 cells were incubated with SP600125 for 1 h. Then, cells were transfected with empty vector, TNC plasmid and/or sic-Jun. After the indicated treatment, gelatin zymography was performed to analyse the MMP activity (E- top); MMP9 mRNA and protein levels were analysed by RT-qPCR (D) and western blot (E- bottom), respectively. Data represent the mean ± SD. (n = 3, *P < 0.05).

Based on our data, TNC induced c-Jun phosph-orylation through activation of JNK. The AP-1 complex consists of c-Jun/c-Fos heterodimers that bind to the consensus DNA sequence 5-TGAG/CTCA-3. AP-1/c-Jun is thought to be a central transcription factor in the regulation of cancer invasion [21, 22]. A c-Jun binding site was found in the *MMP9* promoter sequence (Figure 5A). To observe whether the TNC/c-Jun/MMP9 axis is involved in pancreatic cancer development, we transfected PANC-1 cells with siTNC or TNC plasmid and examined the binding activity of c-Jun to the *MMP9* promoter. The ChIP assay confirmed that c-Jun directly binds to the *MMP9* promoter. We found that downregulation of TNC significantly reduced c-Jun binding activity to the *MMP9* promoter, whereas upregulation of TNC enhanced the DNA binding activity of c-Jun (Figure 5B). To determine the function of c-Jun binding to the *MMP9* promoter in response to TNC regulation, pGL3-MMP9-S1 (-897/+249), pGL3-MMP9-S2 (-314/+249), and pGL3-MMP9-Mut (-897/+249) promoter luciferase constructs were generated. We examined the effect of TNC on the transcriptional activity of *MMP9* using a luciferase reporter assay. As shown in Figure 5C, knockdown of TNC inhibited the pGL3-MMP9-S1 promoter activity. Upregulation of TNC induced transactivation of pGL3-MMP9-S1, whereas the cells treated with SP600125 or sic-Jun dramatically inhibited the TNC-induced *MMP9* transcriptional activity. The activity of pGL3-MMP9-S2 and pGL3-MMP9-Mut were not significantly changed by the above treatment (Figure 5C), indicating that the sequence located between -897/-313 contains the active binding site of c-Jun, which was critical for activation of the *MMP9* promoter. The luciferase reporter assay showed that TNC transactivated the *MMP9* promoter activity by regulating c-Jun activity. And then, we hypothesized that TNC might function by activating c-Jun to increase MMP9 expression. And then, zymography, RT-qPCR and western blotting were performed to test the activity of TNC on MMP9 activity and expression under different conditions, TNC and SP600125/sic-Jun treatments. As shown in Figure 5D and 5E, overexpression of TNC resulted in a significant increase in MMP9 mRNA and protein in PANC-1 cells, whereas the TNC-induced effects were inhibited by SP600125 or sic-Jun. Zymography utilizing gelatin as a substrate for MMP2 and MMP9 revealed that TNC stimulated MMP9 enzyme activity, but the activity was inhibited by SP600125 or sic-Jun (Figure 4E). However, TNC did not affect MMP2 expression. Consequently, the results suggested that TNC regulated MMP9 expression and activity through activation of JNK/c-Jun signalling.

### TNC regulates the phosphorylation of paxillin by modulating JNK/FAK activity

**Figure 6:**
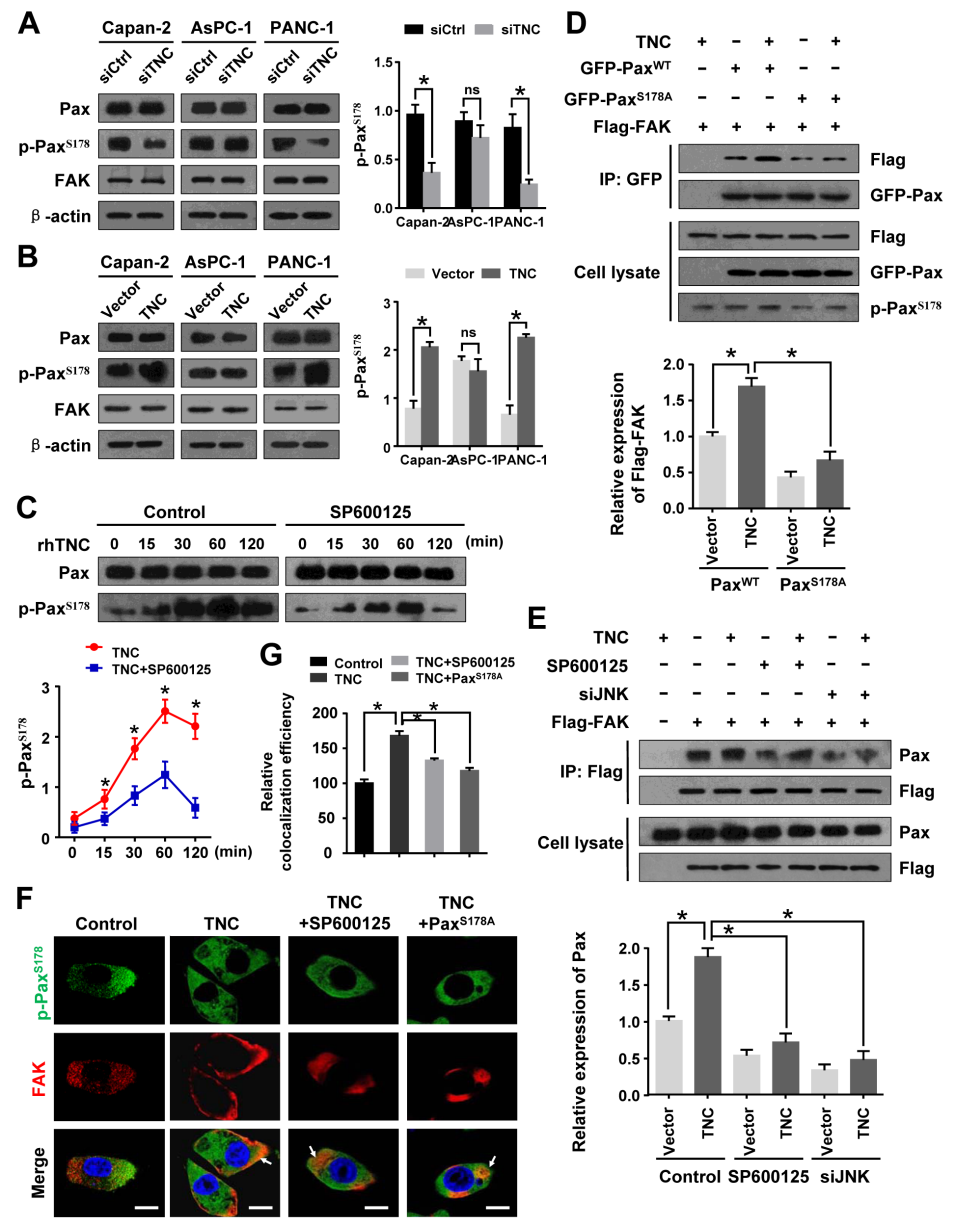
JNK/FAK mediated TNC-induced Paxillin^S178^ phosphorylation **(A)** Western blot analysis of Paxillin, p-Paxillin^S178^ and FAK expression in Capan-2, AsPC-1 and PANC-1 cells transfected with siTNC or siControl. **(B)** The protein levels of Paxillin, p-Paxillin^S178^ and FAK in Capan-2, AsPC-1 and PANC-1 cells with TNC expression plasmid or control vector. **(C)** PANC-1 cells were preincubated with SP600125 or DMSO for 1 h before exogenous TNC stimulation. Cells were harvested at the indicated time, and the protein expression was assessed using antibodies against Paxillin and p-Paxillin^S178^. **(D)** PANC-1-Flag-FAK cells transfected with GFP-Paxillin^WT^ or GFP-Paxillin^S178A^ were treated or not treated with TNC plasmid. Cell lysates were immunoprecipitated with GFP antibody, and immunoblotting was performed with Flag and GFP antibodies. **(E)** After pretreatment with SP00125 for 1 h, PANC-1-Flag-FAK cells were transfected with TNC and/or siJNK followed by immunoprecipitation of the Flag antibody, and immunoblotting was performed with Paxillin and Flag antibodies. **(F)** Confocal microscopy images of PANC-1 cells that were preincubated with SP600125 followed by transfection with TNC and/or Paxillin^S178A^ plasmid. The green signal represents the staining corresponding to p-Paxillin^S178^, the red signal represents FAK, and the blue signal represents the nuclear DNA staining by DAPI. Scale bar, 10 μm. **(G)** Quantify the colacalization efficiency of p-Paxillin^S178^ and FAK. Data represent the mean ± SD.

Several reports have indicated that tumour cells have an active potential to induce cell migration and invasion by activating FAK/Paxillin activity [23, 24]. To further test the influence of TNC on tumour metastasis-related genes, we examined the expression of Paxillin, p-Paxillin^S178^ and FAK. We found that p-Paxillin^S178^ was significantly inhibited by siTNC in Capan-2 and PANC-1 cells, but not AsPC-1 (Figure 6A). We speculate that TNC regulate the migration and invasion of AsPC-1 cells maybe do not dependent on p-Pax pathway. Accordingly, ectopic expression of TNC resulted in an increase in the p-Paxillin^S178^ level in these cells (Figure 6B). Moreover, PANC-1 cells were exposed to exogenous TNC for the indicated time, and the subsequent total Paxillin and p-Paxillin^S178^ were analysed by western blot. It was shown that TNC markedly increased the expression of p-Paxillin^S178^ Paxillin-dependent manner, which indicated that TNC modulates the phosphorylation of PaxillinS178 in pancreatic cancer cells. (Figure 6C-left), In addition, SP600125 markedly reduced the phosphorylation of PaxillinS178 in TNC-stimulated PANC-1 cells (Figure 6C-right), which indicated that JNK activation plays a vital role in TNC-induced activation of Paxillin^S178^ (We also did the knockdown experiment for JNK and found it could decreased p-Paxillin^S178^ expression, data not shown). The p-Paxillin^S178^/FAK complex has been affirmed to contribute to cell migration in a number of different tumour types [17, 24]. To test the effect of TNC on the regulation of the p-Paxillin^S178^ /FAK complex, PANC-1 cells transfected with Flag-FAK and with either GFP-Paxillin^WT^ or a GFP-Paxillin^S178A^ mutant were stimulated with TNC plasmid. Then, the cell lysates were immunoprecipitated with GFP antibody. Overexpression of TNC increased the FAK-Paxillin combination in PANC-1-Paxillin^WT^ cells. However, PANC-1-Paxillin^S178A^ cells exhibited a markedly reduced FAK-Paxillin interaction (Figure 6D). It has been reported that JNK phosphorylation of Paxillin^S178^ is required for the combination of Paxillin with FAK [17, 24]. We further coexpressed TNC and Flag-FAK in PANC-1 cells in the presence or absence of SP600125 or siJNK. After immunoprecipitating Flag, we found that the association of p-Paxillin with FAK was more abundant in TNC overexpressing cells, whereas the effect was inhibited by SP600125 or siJNK (Figure 6E). Therefore, we speculated that TNC/JNK signalling activated Paxillin^S178^ and promoted its combination with FAK. In addition, we tested p-Paxillin and FAK colocalization in the tumour cells by immunofluorescence. The result revealed that TNC promoted Paxillin^S178^ phosphorylation and its colocalization with FAK at the cell periphery. However, in the presence of SP600125 or expression of the Paxillin^S178A^ mutant, TNC’s effects were clearly inhibited (Figure 6F).

### TNC promotes tumour cell migration and adhesion by activating JNK/Paxillin/FAK signalling

**Figure 7:**
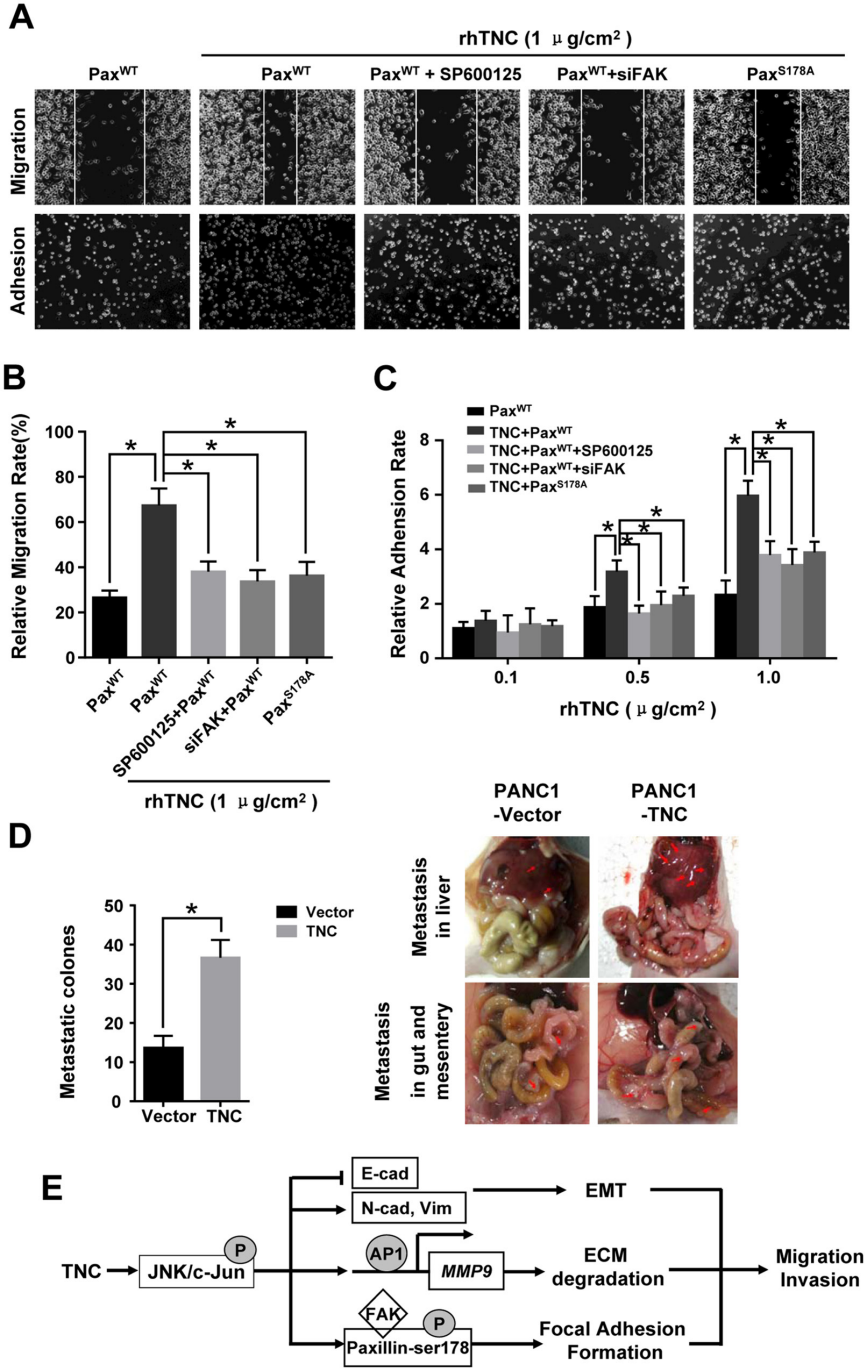
TNC promotes tumour cell migration and adhesion by regulating JNK/Paxillin/FAK signals PANC-1 cells stably expressing Paxillin^WT^, Paxillin^WT^+SP600125, Paxillin^WT^+siFAK or Paxillin^S178A^ were plated into plates coated with rhTNC. **(A)** The images show the migration abilities (top) and adhesion ability (bottom) of the indicated cells onto the TNC coated plates (1 μg/cm^2^). **(B)** The average migratory ratio of indicated cells was statistically analysed from three independent experiments. Data represent the mean ± SD. (*P < 0.05). **(C)** The relative adhesion rates of the indicated cells to the different concentrations of TNC (0.1 μg/cm^2^, 0.5 μg/cm^2^, 1 μg/cm^2^). Data represent the mean ± SD. (n = 3, *P < 0.05). **(D)** Nude mouse intraperitoneal tumor cell metastasis assay. The total numbers of visible metastatic lesions in abdominal cavity are presented. **(E)** A schematic diagram showing the effect of TNC on migration and invasion via activation of JNK signalling.

Paxillin/FAK molecules are vital components in focal adhesion formation, which promotes cell migration and adhesion. Therefore, we examined whether TNC functions on tumour cell aggression and adhesion ability by activating JNK/Paxillin/FAK signalling. Wound healing and adhesion assays were performed. The results indicated that, when grown on TNC-coated plates, cells migrated approximately three times faster than control cells, and the cell adhesion ability was increased. However, a significant delay in the wound closure and a number of adhesive cells were observed in PANC-1-Paxillin^WT^ cells after preincubation with SP600125, which indicated that exogenous TNC induced migration and adhesion by activating JNK molecules. To further examine the role of FAK and Paxillin S178 in TNC-stimulated migration and adhesion potential in PANC-1 cells, cells were stably transfected with PaxillinWT +siFAK or Paxillin^S178A^. The migration and adhesion ability was tested in PANC-1 cells transfected with siFAK or S178A mutant. The results suggested that JNK, FAK and p-Paxillin^S178^ were required for TNC-induced motility and adhesion in tumour cells (Figure 7A and 7B). In addition, cell adhesion function was affected in a dose-dependent manner on TNC-coated plates (Figure 7C).

Furthermore, the nude mouse intraperitoneal tumor metastasis experiments showed that injection of PANC-1 cells with TNC overexpression induced the numbers of abdominal metastasis nodules (Figure 7D) These data indicate that TNC is essential in pancreatic cancer development and metastasis.

## DISCUSSION

It has been reported that TNC is overexpressed in pancreatic cancer invasive fronts and is clinically associated with metastasis and poor prognosis [7, 8, 13]. Previously, we demonstrated that TNC promoted tumour cell development and apoptosis resistance in pancreatic cancer [7, 25]. In the present study, we determined that TNC was essential to trigger migration and invasion in pancreatic cancer both *in vitro* and *vivo*. Consistent with previous findings, we showed that TNC may act as an endogenous protein in the tumour microenvironment to initiate metastatic processes in pancreatic cancer. Therefore, the expression of TNC might be a clinically effective indicator for distinguishing malignancy, as well as for identifying malignant tumour metastasis. In addition, we further revealed the molecular mechanism for TNC modulation of tumour cell motility. To date, several molecules have been reported to influence TNC activity. TNF-α in the tumour microenvironment induces TNC expression in hepatocellular carcinoma (HCC) cells through the NF-κB pathway and then promotes HCC migration [26]. TNC induced activation of ERK1/2, JNK and p38 MAPK signalling in airway remodelling in asthma [10]. Co-expression of MMP9, MMP2 and TNC contributes to pancreatic cancer progression [13, 14]. Also, our previous immunohistochemistry study demonstrated that higher expressions of JNK/p-JNK and Paxillin/p-Paxillin S178 in tumor tissues were found when compared with those in the paracarcinoma tissue [27]. Based on these clinical studies, we speculated that there would be the positive correlation among TNC and these molecules. However, it remains to be understood which signalling molecules and pathways are involved in TNC control of metastasis in PDAC. In the present study, according to the biological cascade of tumour metastasis, we determined that an intrinsic affiliation among the cancer cell EMT programme, ECM degradation, focal adhesion formation and activation of related signalling molecules was involved in TNC’s function in pancreatic cancer metastasis.

TNC could be synthesized by the cancer cells and then efficiently secreted in the stroma [7]. Integrins are heterodimeric transmembrane receptors that connect extracellular matrix proteins to numerous intracellular pathways can regulate cell adhesion, differentiation, migration, invasion and metastasis [28]. Katoh et al. reported that integrin ανβ1 and ανβ6 act as TNC receptors, constituting a signaling pathway to evoke EMT in breast cancer cells [29]. JNK is highly expressed and active in metastasis of PDAC [11, 12], which is one of integrin-associated signalling events [28, 30]. However, the relationship of the integrins to signalling events during TNC-mediated migration and invasion still needs to be verified by further study. The oncogenic functions of JNK are primarily based on its ability to phosphorylate c-Jun and to activate AP-1 in response to a plethora of extracellular stimuli. In this study, our data provides evidence that TNC leads to increased phosphorylation of JNK/c-Jun in pancreatic cancer cells. In addition, the TNC-induced phosphorylation of JNK/c-Jun and the cell migration/invasion activity was reduced when JNK was inhibited by the specific inhibitor SP600125. We suggest that JNK activation might be a key event in TNC-mediated motility in pancreatic cancer cells. Based on the molecular complexity of the JNK signalling pathways involved in cancer metastasis and development, several metastasis-associated targets were detected in the present study, including the EMT markers, MMP2/9 and Paxillin/FAK.

Development of EMT involves many morphological and molecular features and plays an important role in PDAC invasion and metastasis [15]. Indeed, changes in the expression level of EMT markers, including E-cadherin, ZO-1, N-cadherin and Vimentin, have often been used to monitor the progress of EMT. It has been shown that TNC can induce EMT-like changes, including loss of intercellular adhesion and enhanced migration in breast cancer cells [16]. Moreover, TNC mediates ZO-1 degradation via MAPK signalling in tissue development [31]. Previous studies indicate that JNK/c-Jun increase migration and invasion in various cancer cells primarily by inhibiting E-cadherin expression, which is a major event and a key biomarker for EMT [32-34]. In this study, we indicated that overexpression of TNC has a significant effect on inducing EMT via increased phosphorylation of JNK and c-Jun in pancreatic cancer. We found that ectopic expression of TNC in pancreatic cancer cells resulted in downregulation of the epithelial marker E-cadherin and concomitant downregulation of the mesenchymal markers N-cadherin and Vimentin, whereas SP600125 significantly reversed the TNC-induced change in EMT markers. Thus, overexpression of TNC most likely leads to increased metastatic potential by promoting EMT, and this process is required for activation of JNK signalling. The EMT process is now considered to occur at the early adenoma stage. In addition, EMT progression due to the change to embryonic morphogenesis and molecular phenotypes then in response to a combination of extracellular signals in the tumour microenvironment initiate the tumour cell motility [35]. Thus, this indicates that TNC initiated a metastatic cascade by inducing the cancer cell EMT programme. High expression of TNC in the early stage of cancer might be a predictor for clinical evidence of carcinoma cell EMT events in pancreatic cancer.

Paxillin is a multidomain adaptor protein that recruits a number of signal transducers to focal adhesions, where it mediates the transduction of signals from integrins and growth factors to downstream regulators of cell migration. Notably, the Paxillin^S178^ activated by JNK acts as a primary mechanism required for focal adhesion formation, cell adhesion and cell migration. The expression of both TNC and Paxillin were increased in tissues of squamous cell carcinoma with lymph node metastasis [36]. Therefore, we detected whether JNK-mediated phosphorylation of Paxillin^S178^ may perform a role in TNC-related PDAC metastasis. We found that the p-Paxillin^S178^ was stimulated by TNC with a similar time course as that of JNK activation. FAK must be localized to cellular focal adhesions to induce efficient phosphorylation of Paxillin on tyrosine 31 and 118, leading to recruitment of a number of signalling molecules, and thus regulating focal adhesion dynamics and cell migration [37, 38]. In the present study, we found that JNK activation is required for the association between Paxillin and FAK, and this association is apparently dependent on phosphorylation of serine 178 on Paxillin [17, 24, 37]. Moreover, TNC markedly activated JNK to induce the phosphorylation of Paxillin^S178^, and in turn increased the assembly of Paxillin and FAK. In addition, it has been shown that TNC/JNK-dependent cell migration and adhesion requires phosphorylation of Paxillin^S178^. Therefore, we indicated that TNC regulated tumour cell motility by regulating focal adhesion function via activation of multiple signalling pathways, namely JNK/Paxillin/FAK. This study showed a novel mechanism in which coordination of the interaction of multiple molecules regulated by TNC generated specific biological activities. These results provide evidence that focal adhesion-related molecules might be gene targets for inhibiting tumour cell metastatic processes, especially in pancreatic cancer tissue with increased deposition of TNC.

MMPs mediate homoeostasis of the extracellular environment. Several MMPs are regulated by TNC, such as MMP1/13, MMP2/9 and MMP3. The pattern of TNC-induced MMP expression differs in tissue, which might constitute a variable regulation effect on tissue function [10, 39]. It was reported that TNC expression was correlated with the activation of MMP2 in a co-culture of pancreatic cancer BxPC-3 cells with stromal fibroblasts [40]. In addition, clinical evidence has shown that co-expression of MMP9 and TNC contributes to pancreatic cancer progression [13]. MMP2 and MMP9 are type IV collagenases secreted into the cell-matrix interface where they degrade type IV collagen in both the intercellular matrix and the basal membrane. They perform a key role in cancer metastatic processed by paving the way for cancer cell invasion. Thus, we observed that TNC functions in the regulation of the expression and activity of type IV collagenases in pancreatic cancer cells. We discovered that TNC increased MMP9 activity through JNK/AP-1 transactivation of its expression, but not MMP2, which might be because MMP2 promoter regions do not contain AP-1 binding sites. Therefore, the mechanism by which TNC and MMP2 were co-overexpressed in pancreatic cancer tissue needs to be further investigated. Our data confirm that JNK/c-Jun-mediated transcriptional activation of MMP9 may account for the higher metastatic potential under TNC stimulation. Therefore, TNC promoted cancer cell migration and invasion both by altering the tumour environment and by transforming the biological features of cells. This modulated the metastatic process in the form of a biological cascade effect.

In conclusion, the present observations confirmed that TNC efficiently induces pancreatic cancer cells to undergo EMT progression, degrade the ECM through secretion of activated MMP9, and regulate focal adhesion formation via JNK/c-Jun signalling activation, by which tumour cell invasion and migration was enhanced. TNC mediated metastasis and adhesion of PDAC through altering the tumour environment and transformation of cellular biological features. Thus, TNC might act as a potential therapeutic target and predictor for pancreatic cancer metastasis at an early stage (Figure 7E).

## MATERIALS AND METHODS

### Cell culture

The human pancreatic cancer cell lines Capan-2, AsPC-1 and PANC-1, each with different biological characteristics and metastatic potential, were purchased from the American Type Culture Collection (ATCC). Cells were cultured in RPMI 1640 (Capan-2 and AsPC-1) or DMEM (PANC-1) supplemented with 10% fetal bovine serum (FBS; Biological Industries, Kibbutz Beit-Haemek, Israel) at 37 °C with 5% CO_2_. For the induction experiments, PANC-1 cells were treated with 5 μg/ml recombinant human TNC (rhTNC; Millipore, Billerica, MA, USA) for the indicated time. The inhibitor of JNK SP600125 (Selleck Chemicals, Houston, TX, USA) was applied to the culture medium at a final concentration of 20 μM.

### Plasmids, small interfering RNA, and transfection

Human TNC cDNA was amplified from a pBS-HxB.L (Addgene plasmid #65414) plasmid and then cloned into a pcDNA3.1 vector (Invitrogen, Gaithersburg, MD, USA). *MMP9* promoter regions from -897 to +249 (containing a candidate c-Jun binding site) or from -314 to +249 (lacking the candidate c-Jun binding site) relative to the transcription start site (TSS) were amplified from human genomic DNA by PCR. And then the fragment were cloned into the Kpn I and Xho I restriction sites in the luciferase reporter pGL3-Basic vector (Promega, Madison, WI, USA), namely pGL3-MMP9-S1 or pGL3-MMP9-S2. The *MMP9* promoter mutated luciferase construct (pGL3-MMP9-Mut) were constructed by the Fast Mutagenesis System (TransGen Biotech, Beijing, China). The pRK-GFP-Paxillin and pWZL-Neo-Myr-Flag-PTK2 plasmids were a gift from Kenneth Yamada (Addgene plasmid #50529) and William Hahn & Jean Zhao (Addgene plasmid #20610), respectively. The expression vector GFP-Paxillin^WT^ encoding a Paxillin mutant with a serine 178 to alanine mutation (GFP-Paxillin^S178A^) was constructed by the Fast Mutagenesis System (TransGen biotech). Three small interfering RNAs (siRNAs) targeting independent sequences of human TNC (siTNC), c-Jun (sic-Jun), JNK1 (siJNK, JNK mentioned in the article is JNK1) and FAK (siFAK) genes were designed and synthesized by GenePharma (Shanghai, China). We tested the mRNA or protein levels for confirming the interference efficiency of siRNA after the pancreatic cancer cells transfected with three independent siRNA. The siRNA displaying optimal knockdown efficiency was selected for further experiments (Supplementary Figure 1). Non-targeting siRNA was used as a control (siControl). The transfection of cells with plasmids or siRNAs was performed using Lipofectamine 2000 Reagent (Invitrogen) according to the manufacturer’s protocol, for example, for 6-well plates 4 μg plasmid DNA was used and the final concentration of the siRNA is 80 nM.

### Cell migration and invasion assay

The migration and invasion abilities of pancreatic cancer cells were evaluated using non-Matrigel-coated and Matrigel-coated Transwell inserts (BD Biosciences, San Diego, CA, USA), respectively. Briefly, 5×10^4^ cells in 500 μl of serum-free medium were added to the upper chamber, and the medium containing 10% FBS was added to the lower chamber. The cells were left to invade the Matrigel-coated for 48 h, and non-Magrigel-coated for 24 h. The non-invading cells on the upper surface of the membrane were removed by wiping, and the invading cells were fixed and stained with crystal violet. The number of migrating or invading cells was counted under a microscope in five predetermined fields for each membrane at ×200 magnification.

### Western blot

Cultured cells were collected and solubilized using protein lysis buffer. The proteins were then separated by size using SDS-PAGE and transferred to polyvinyl difluoride membranes (Millipore). The membranes were incubated with primary antibodies followed by secondary antibody (Santa Cruz Biotechnology, Santa Cruz, CA, USA). The immunoreactive proteins were detected using an enhanced chemiluminescence kit (Millipore). The primary antibodies were rabbit anti- E-cadherin, rabbit anti-N-cadherin, rabbit anti-Vimentin (Cell Signaling Technology, Danvers, MA, USA), mouse anti-MMP9, mouse anti-MMP2, mouse anti-JNK1, mouse anti-p-JNK, rabbit anti-c-Jun, mouse anti-p-c-Jun, mouse anti-FAK, mouse anti-β-actin (Santa Cruz Biotechnology), rabbit anti-Paxillin, and rabbit anti-p-Paxillin (Abcam, Cambridge, MA, USA). The digital images of the Western blotting bands were quantified by Meta Morph software (MDS Analytical Technologies) after the background subtraction.

### Immunofluorescence

Coverslips were coated with or without rhTNC/BSA. Uncoaded proteins were allowed to be adsorbed overnight at 4 °C, wells were then washed twice with PBS and sterilized by UV exposure for 20 min. The PANC-1 cells were seeded on coverslips, cultured for 48 h, and then fixed with 4% paraformaldehyde. After washing with PBS, the cells were permeabilized with 0.25% Triton X-100 for 10 min, washed with PBS, blocked with 3% goat serum blocking solution, and then incubated with primary antibodies overnight at 4 °C. The cells were then washed with PBS and incubated with fluorescein isothiocyanate-conjugated secondary antibody (Santa Cruz Biotechnology). Nuclei were stained with DAPI. Confocal microscopy studies were per-formed with a laser scanning microscope (TCS-SP2-AOBS-MP, Leica Microsystems CMS, Wetzlar, Germany). Each image was obtained using identical microscope settings, including laser power, gain, and contrast.

Images were semiquantitatively analyzed using ImageJ software (www.imageJ.nih.gov/ij/). Briefly, >50 cells per substrate and protein type were outlined, and the average flurescence intensity within each cell was measured. An area next to the cell with no fluorescence was also measured, and this value was subtracted from the average fluorescence intensity for that cell to correct for any background intensity. In addition, the area of p-Pax^S178^ that overlapped with FAK spots was then determined in percent. The average area of p-Pax^S178^ that overlapped with FAK in control cells was defined as 100%, and the degree of overlap in experimental groups were calculated relative to this value.

### RT-qPCR

Total RNA was isolated from the cultured cells using an Eastep Total RNA Extraction kit (Promega) according to the manufacturer’s protocol. Then, total RNA was reverse transcribed using a TransScript First-Stand cDNA Synthesis kit (TransGen Biotech). RT-qPCR was subsequently performed with TransStart Green qPCR SuperMix (TransGen Biotech), and products were detected with a DA7600 Real-time Nucleic Acid Amplification Fluorescence Detection System (Bio-Rad, Hercules, CA, USA). We quantified the transcripts of the housekeeping gene glyceraldehyde 3-phosphate dehydrogenase (GAPDH) as an internal mRNA quantity control. All primers for cDNA amplification of various target genes were designed and optimized using Oligo 7.0 software (Molecular Biology Insights, West Cascade, USA) and synthesised by Sangon Biotech (Shanghai, China). The relative expression levels of the target genes were calculated by normalizing the cycle threshold (Ct) values of the target gene to the Ct values of GAPDH (ΔCt) and determined as 2^-ΔCt^. The RT-qPCR products were subjected to electrophoresis.

### Chromatin immunoprecipitation assay

A chromatin immunoprecipitation (ChIP) assay was performed using a ChIP kit (Millipore) according to the manufacturer’s protocol. Anti-c-Jun antibody was used for immunoprecipitation to enrich the promoter fragments containing putative c-Jun binding sites of target genes in PANC-1 cells endogenously expressing c-Jun. The isotype IgG was used as a negative control. The primers for amplification of the *MMP9* promoter region containing a c-Jun putative binding site from -537 to -530 relative to the TSS were as follows: 5’-CTACTGTCCCCTTTACTGC-3’ (forward) and 5’-AGATATCCTCCCCAAACCC-3’ (reverse).

### Dual-luciferase reporter assay

Cells were seeded into 24-well plates, cultured without antibiotics and grown to 80% confluence. Then, cells were incubated with SP600125 or DMSO for 1 h. Subsequently, siTNC, TNC, TNC+sic-Jun as well as their controls were cotransfected with pGL3-Basic, pGL3-MMP9-S1, pGL3-MMP9-S2 or pGL3-MMP9-Mut and the internal control pRL-TK into the cells. After 48 h, the luciferase activities of the cells were measured using a dual-luciferase reporter assay kit (Promega). Reporter luciferase activity was normalized to Renilla luciferase activity.

### Gelatin zymography

Cells were pretreated with SP600125 or DMSO for 1 h. Then, cells were transfected with empty vector, TNC plasmid and/or sic-Jun, separately. After incubation, we collected the media supernatant and performed gelatin zymography to check the MMP activity. Subsequently, equal amounts of protein from the samples were loaded onto 10% SDS-PAGE gels containing 0.1% (w/v) gelatin (Sigma-Aldrich, St Louis, MO, USA) in the absence of reducing reagent. After electrophoresis, the gels were washed twice in 2.5% (w/v) Triton X-100 for 40 min at room temperature and incubated in 50 mM Tris-HCl (pH 7.6), 200 mM NaCl, 5 mM CaCl_2_, and 0.02% (w/v) Brij35 for 72 h at 37 °C under gentle agitation. The gels were finally stained with 0.5% Coomassie Brilliant Blue R-250 (Sigma) for 3 h and destained with 30% methanol and 10% glacial acetic acid. Gelatinolytic activity is shown as clear areas in the gel.

### Immunoprecipitation

Cell lysates were incubated with protein A/G-agarose beads (Millipore) for 1 h at 4 °C to minimize nonspecific binding. Lysates were transferred to clean tubes and incubated further with the appropriate antibody, including rabbit anti-GFP or rabbit anti-Flag (Sigma). After incubation at 4 °C overnight, protein A/G-agarose beads were added and rotated for 2 h. The complexes were washed three times with 1× immunoprecipitation buffer. Proteins were eluted by boiling in 2× SDS loading buffer and subjected to western blot using GFP, Flag, p-Paxillin, and Paxillin antibodies.

### Wound-healing assay

PANC-1 cells were seeded in TNC-coated plates and allowed to grow into a monolayer. A linear wound was made in the cell monolayer with a sterile pipette. For the next 24 h, cells were serum starved, and the photomicrographs were taken of live cells at ×100 magnification. The relative closed-wound distance was calculated after measuring the width of at least four wounds.

### Adhesion assay

PANC-1 cells were seeded in TNC-coated (0.1 μg/cm^2^, 0.5 μg/cm^2^, 1 μg/cm^2^) 24-well plates. The nonadherent cells were counted after being cultured for 3 h, and the percentage of adherent cells was calculated as (1 — non-adherent cells/total inoculated cells) ×100%.

### Animal experiments

A nude mouse experiment was performed to assess *in vivo* tumour metastasis ability of PANC-1 cells stably expressing TNC or empty vector. These cells (5×10^6^ cells) were inoculated into the abdominal cavity of 5-week-old BALB/c athymic nude mice (Beijing HFK Bio-Technology, Beijing, China), eight mice per group. All animal studies were approved by the Ethics Committee of the Tianjin Medical University Cancer Institute and Hospital and conducted by skilled experimenters under an approved protocol in accordance with the principles and procedures outlined in the NIH Guide for the Care and Use of Laboratory Animals. Numbers of visible metastases in the peritoneum, gut, mesentery and liver, as well as invisible micrometastases, were counted after mice were sacrificed [41].

### Statistical analysis

Data are presented as the mean ± standard deviation (SD). Student’s *t*-test and ANOVA was used to compare differences between the experimental group and the control group. Statistical significance was defined as *P* < 0.05.

## SUPPLEMENTARY MATERIALS FIGURE



## Abbreviations

TNC: Tenascin-C
PDAC: pancreatic ductal adenocarcinoma
ECM: extracellular matrix
EMT: epithelial-mesenchymal transition
PSCs: pancreatic stellate cells
JNK: c-Jun N-terminal kinase
MAPK: mitogen-activated protein kinase
EGF: epidermal growth factor
TNF: tumour necrosis factor
TGF-β: transforming growth factor β
AP-1: activator protein-1
MMPs: matrix metalloproteinases
ATCC: American Type Culture Collection
siRNA: small interfering RNA
RT-qPCR: reverse transcription-quantitative PCR
ChIP: chromatin immunoprecipitation
