# Supplementary material for: Tenascin-C induces migration and invasion through JNK/c-Jun signalling in pancreatic cancer
Source: Oncotarget. 2017 Aug 10;8(43):74406–22. doi: 10.18632/oncotarget.20160 (PMC5650351; doi:10.18632/oncotarget.20160)
Supplement: Supplementary file 1 [file oncotarget-08-74406-s001.pdf]

# Tenascin-C induces migration and invasion through JNK/c-Jun signalling in pancreatic cancer

## SUPPLEMENTARY MATERIALS

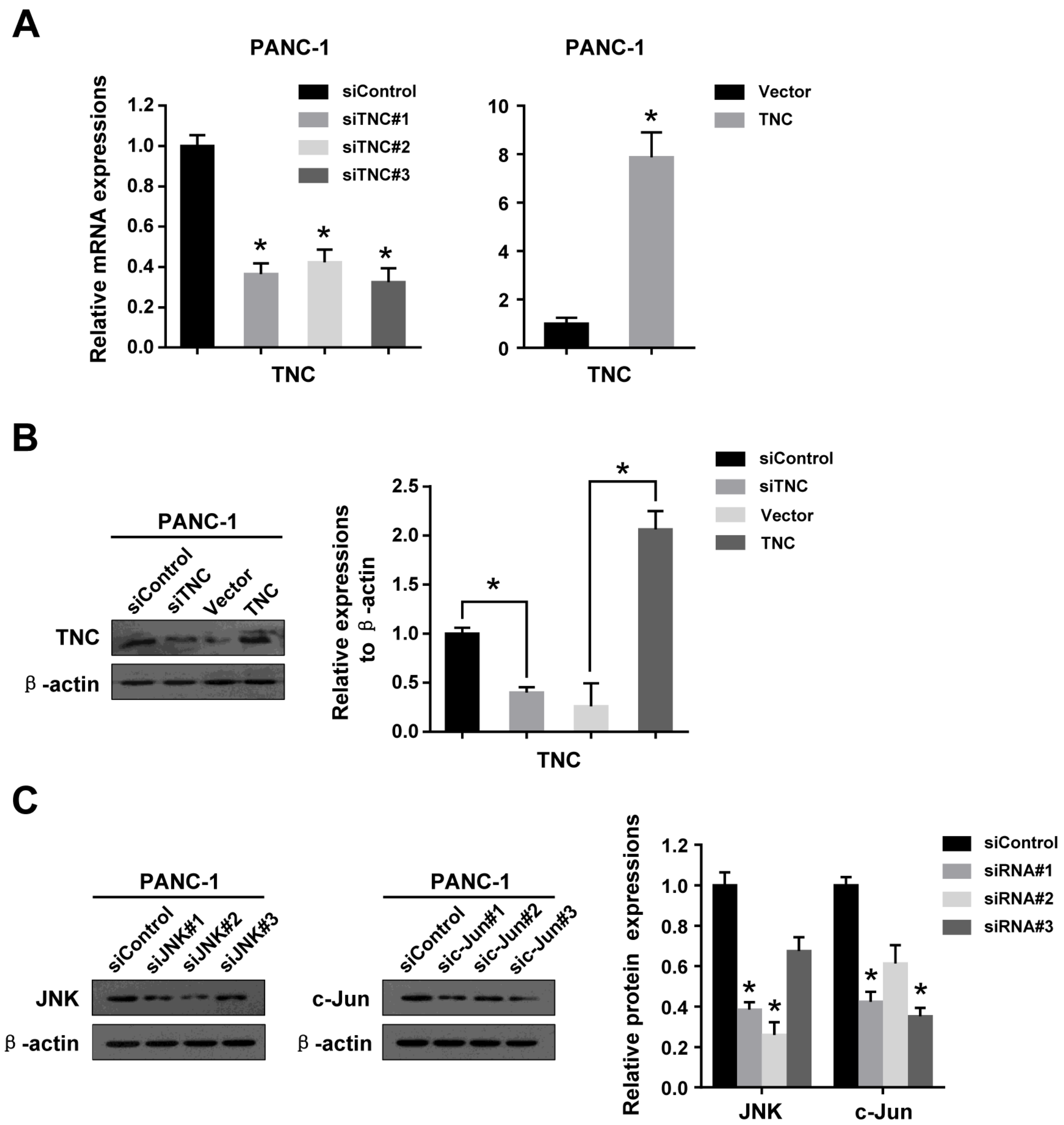


**Supplementary Figure 1: The expression levels of TNC, JNK and c-Jun in PANC-1 cells.** **(A)** the mRNA levels of TNC after the PANC-1 cells were transfected with siTNC or TNC expression plasmid. **(B)** the TNC protein levels in culture supernatants collected from the indicated PANC-1 cells with different conditions were detected by western blot. **(C)** Western blot analysis was performed to test the expressions of JNK and c-Jun in the PANC-1 cells transfected with three independent siRNA or siControl. Data represent the mean ± SD. (n = 3, *$P < 0.05$).
